# Effects of Rhythmic Auditory Stimulation Using Sensory Feedback-Based Wearable Devices on the Gait and Balance in Patients with Parkinson’s Disease: A Systematic Review and Meta-Analysis

**DOI:** 10.3390/brainsci16040359

**Published:** 2026-03-26

**Authors:** Ju-Hak Kim, Myoung-Ho Lee, Myoung-Kwon Kim

**Affiliations:** 1 Department of Physical Therapy, Graduate School, Daegu University, Jillyang, Gyeongsan 712-714, Gyeongbuk, Republic of Korea; rlawngkr2000@naver.com (J.-H.K.); hotayaaa@gmail.com (M.-H.L.); 2 Department of Physical Therapy, College of Rehabilitation Sciences, Daegu University, Jillyang, Gyeongsan 712-714, Gyeongbuk, Republic of Korea

**Keywords:** Parkinson’s disease, rhythmic auditory stimulation, gait, balance, wearable device

## Abstract

Background: This paper presents a systematic review and meta-analysis to identify the effects of Rhythmic Auditory Stimulation (RAS) delivered via wearable devices on the gait and balance in patients with Parkinson’s disease. Method: The PICO criteria were established according to the PRISMA 2020 guidelines, and literature searches were performed across five databases covering studies published between 2015 and 2025: PubMed, Embase, Cochrane, Scopus, and Web of Science. After applying the inclusion criteria, eleven randomized controlled trials (RCTs) were selected. The quality of the studies was evaluated using the PEDro Scale and ROB-2. Statistical analyses were performed using Review Manager 5.4 based on the number of samples, means, and standard deviations to calculate the effect sizes. Result: The analysis results showed that wearable RAS significantly improved the gait speed (SMD = 0.49, *p* < 0.05) and balance ability (SMD = 0.40, *p* < 0.05), while no significant differences in the gait pattern, FOG-Q, or UPDRS-III were observed. The heterogeneity among studies was low, and the funnel plots were distributed symmetrically, indicating minimal publication bias. The average PEDro score was 7.33, suggesting moderate-to-high methodological quality. Conclusion: wearable RAS was identified as an evidence-based intervention effective in improving the gait speed and balance in patients with Parkinson’s disease.

## 1. Introduction

Parkinson’s disease (PD) is a chronic neurodegenerative disorder that occurs primarily in older adults aged 60 years and above. It affects approximately 6 to 10 million older individuals worldwide, and its prevalence continues to increase as the population ages [1,2]. The representative motor symptoms of patients with PD include resting tremor, bradykinesia, rigidity, freezing of gait, and postural instability; non-motor symptoms, such as cognitive impairment, depression, and other psychological and psychiatric problems, are also observed [3]. These characteristics cause substantial difficulties with the activities of daily living, and independent living eventually becomes impossible as PD progresses, leading to a marked decline in the quality of life [4]. Substantial medical treatment costs are incurred at the individual and household levels, increasing the overall socioeconomic burden associated with the disease [5].

PD patients experience impairments in gait and balance. The gait in individuals with PD is characterized by a slowed gait speed, reduced step length, diminished arm swing, difficulties with gait initiation and termination, turning, and freezing of gait [6,7,8]. These problems tend to increase in frequency and duration as the disease progresses [6]. Such symptoms are reported in approximately 50% of PD patients with more than 10 years’ duration [9] and in up to 80% of PD patients [10]. These gait impairments are also a major cause of falls [6]. Abnormal gait results primarily from a dysfunction of the basal ganglia–cortical loop, which interferes with the generation of automated movements [11]. In particular, impairments in internal timing make it difficult to generate movement rhythms, leading to an increased reliance on external cues or external stimulation [12].

Postural instability in PD patients becomes more pronounced as the disease progresses. Although balance is normally maintained through the integration of visual, vestibular, and proprioceptive inputs [13], PD patients exhibit impairments in these sensory systems [14]. In addition, delayed postural responses, difficulties in regulating muscle tone, and impairments in weight shifting contribute to balance loss [15]. Sudden perturbations, bradykinesia, and impaired regulation of muscle contraction force are also factors that compromise postural stability [16]. These deficits arise from a combination of impaired sensory integration, abnormal postural reflexes, and delayed motor execution, with a dysfunction of the basal ganglia leading to deficits in sensory information processing and delays in the generation of automatic postural responses [17]. Furthermore, cognitive decline and reduced attentional capacity in PD patients lead to difficulties in maintaining gait and balance during dual-task conditions. This also explains why conscious attention and cognitive engagement are important components of rehabilitation training [18].

Overall, gait and balance impairments caused by reduced attention, freezing of gait, and postural instability occur in conjunction with deficits in muscle strength, flexibility, postural awareness, and concentration, as well as impairments in sensory processing, muscle tone regulation, muscular responses, and complex postural control. These factors help increase the risk of falls [13,14,19]. When falls occur, subsequent fractures, increased medical costs related to treatment, and deterioration in the quality of life impose significant individual and societal burdens and represent a major cause of increased mortality in older adults because of secondary complications [20]. Gait and balance problems are closely related in terms of their underlying causes and treatment approaches, and training programs aimed at improving the gait and balance abilities are more effective when implemented in parallel [21].

Rehabilitation techniques aimed at addressing the balance and gait impairments in PD patients are currently under active investigation. A variety of rehabilitation approaches are used with the goal of physical training, including functional training that combines sensory training for balance maintenance with visuospatial cognitive abilities and coordination training of muscle strength and endurance, as well as postural control training, functional training, balance training, and gait training [22]. Traditional physical therapy interventions, such as balance training and resistance exercise, as well as functional gait training, treadmill training, and general physical training, are used for these purposes [23]. More recently, interventions incorporating multidisciplinary approaches and advanced technologies have been increasingly emphasized, moving beyond simple repetition, such as dual-task training that includes cognitive or attentional tasks, training based on sensory stimulation, and wearable-based training [24,25].

Rhythmic Auditory Stimulation (RAS) is a neurorehabilitation intervention that improves the gait speed, step length, and rhythm by providing stimulating and repetitive external auditory cues such as music or a metronome. By providing external rhythmic cues to PD patients with impaired internal timing control, this method compensates for deficits in motor planning and execution and serves as an effective gait training approach that regulates gait movements and synchronizes temporal spacing [26]. Clinically, several studies have reported effects such as increased gait speed, reduced freezing of gait, and decreased fall risk [27]. Recently, RAS systems combined with wearable devices have been developed and are being used in various environments [25]. Although conventional free-field RAS can also be applied with relatively few temporal and spatial constraints, wearable device-based delivery may provide additional advantages in terms of portability, real-time individualized adjustment, and continuous monitoring across hospital, home, and community settings [25].

Wearable devices are body-mounted devices based on sensors such as accelerometers and gyroscopes, including inertial measurement units (IMUs). These devices can recognize gait patterns and support motor learning by converting movement into real-time data for collection and analysis [28]. Recently, they have been used as equipment for gait and balance training, providing individualized rhythmic cues or vibrotactile stimulation and functioning as therapeutic devices [29]. In addition, wearable device-based RAS techniques lack temporal and spatial constraints, allowing training to be conducted in hospitals, at home, and outdoors. In addition, they can be worn in community settings, making them increasingly recognized as continuous and highly accessible rehabilitation methods [28].

Randomized controlled trials (RCTs) using RAS techniques have been continuously conducted in patients with central nervous system disorders, including PD, stroke, and spinal cord injury. These include the following: studies showing improvements in gait speed, step length, and cadence in PD patients [30]; studies showing enhanced balance ability and gait symmetry when treadmill training combined with RAS was applied to patients with stroke [31]; and studies reporting improvements in gait speed, step length, and cadence following the application of RAS in patients with a spinal cord injury [32]. Systematic reviews have verified the effectiveness of RAS. Many meta-analyses have reported statistically significant effects of RAS [33,34]. RCTs examining wearable device-based RAS techniques, which have been the focus of recent research, have also been reported [35]. Various RCTs using sensor- and smartphone application-based RAS techniques, as well as glasses-type and leg-mounted devices, are currently being evaluated, providing diverse outcomes across different settings [36,37]. Related systematic review articles also exist and similarly present the introduction and outcomes of various wearable devices [25]. Nevertheless, many studies on wearable device-based RAS mix therapeutic and assessment purposes, indicating a need for a clear distinction. Moreover, no study has comprehensively compiled RCT-based research, and meta-analyses focusing on the therapeutic use of wearable devices remain insufficient, resulting in limited evidence regarding their effects on balance and gait.

Therefore, this study identified RCTs that used wearable device-based RAS techniques in PD patients, organized and analyzed intervention protocols, and objectively verified the effects on gait and balance using statistical methods. Through this verification, this study aimed to obtain objective evidence for a next-generation rehabilitation approach for PD patients and contribute to its adoption as a widely used training method. We hypothesized that wearable device-based RAS would have positive effects on gait parameters and balance ability in individuals with Parkinson’s disease.

## 2. Materials and Methods

### 2.1. Research Design and Procedure

This study quantitatively evaluated the effects of RAS combined with wearable devices on gait and balance in PD patients using a meta-analysis approach. In accordance with the PRISMA 2020 guidelines, the study was conducted through PICO formulation, search strategy development, study selection criteria, quality assessment of the included studies, and statistical analysis. All procedures were reviewed and conducted through discussion and consensus by two researchers holding doctoral degrees in physical therapy. EndNote 21 and Microsoft Excel 365 were used for reference management.

The study protocol was prospectively registered in the International Prospective Register of Systematic Reviews (PROSPERO), an international database for systematic reviews, under the registration number CRD420251070771. The review protocol is publicly accessible via the PROSPERO database. No amendments were made to the registered protocol. The PICO criteria were defined as follows [38]:

(1) PICO formulation

Population (P): Patients diagnosed with PD;

Intervention (I): Rhythmic auditory stimulation provided based on wearable devices;

Comparison (C): Randomized control groups, including no-intervention controls, usual care controls, or other types of control interventions;

Outcome (O): Gait-related parameters (e.g., gait speed, step length, cadence) and balance-related parameters (e.g., Berg Balance Scale [BBS] and Timed Up and Go [TUG]).

### 2.2. Information and Search Strategy

For the literature search, studies published from 1 January 2015 to 15 May 2025 were identified in five databases: PubMed, Embase, the Cochrane Library, Scopus, and Web of Science. The search was limited to studies published from 2015 onward to focus on recent developments in wearable sensor technology and digital rehabilitation systems.

Using the advanced search functions of each database, the search included all terms referring to PD patients (Parkinson, Parkinson’s, Parkinsonism, and Parkinson*); all terms related to wearable devices (wearable device, inertial measurement unit, IMU, gyroscope, digital feedback, and smart*); all terms related to rhythmic auditory techniques (rhythmic auditory stimulation, RAS, rhythmic auditory cue*, auditory feedback, and metronome); and all terms related to gait and balance (gait, walk, locomotion, balance, and motion

The primary search terms were Parkinson, rhythmic auditory stimulation, wearable device, gait, and balance. In constructing the search queries, OR and AND operators were used within and across databases to combine and include relevant terms. In addition, searches were conducted by limiting the fields to the title and abstract (e.g., Title/Abstract or ti/ab) in each database. The asterisk (*) was used as a truncation symbol to capture multiple word endings and variations of the search terms. The full search strategies for each database are provided in Supplementary Material (Supplementary Tables S1–S5).

**Figure 1:**
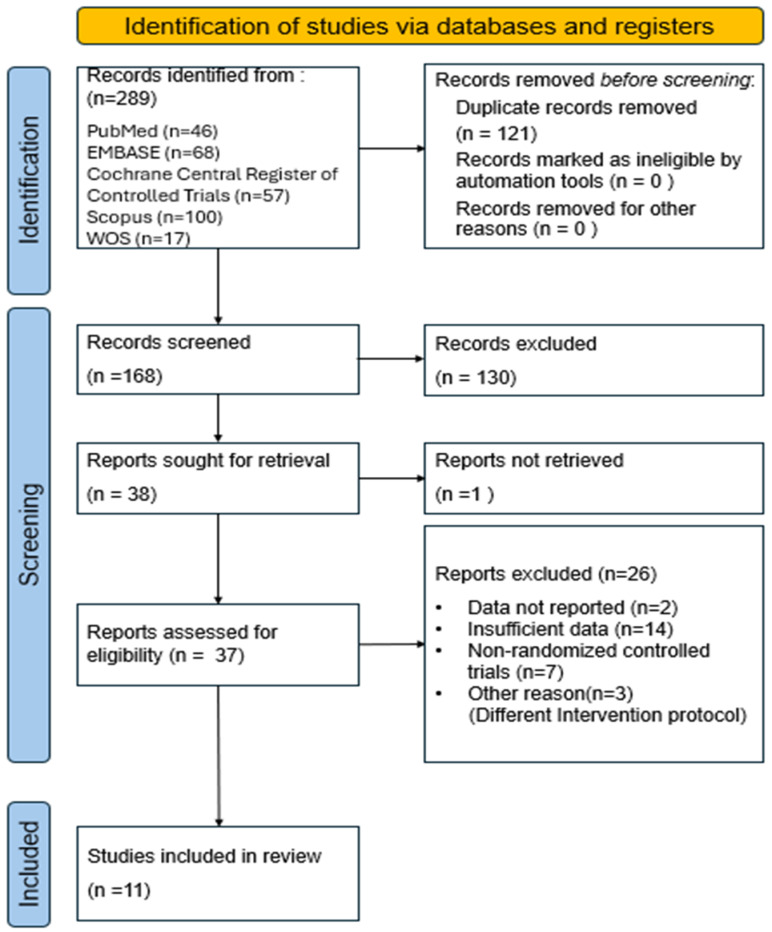
PRISMA flow chart.

In addition, all processes of searching, screening, and selecting studies were visualized using a PRISMA flow diagram (Figure 1). Two hundred and eighty-nine records were identified from the five databases. After removing duplicate records, 168 studies remained. Thirty-eight studies were selected after title screening and an abstract review. One study could not be retrieved in full text, resulting in 37 full-text articles assessed for eligibility. Subsequently, two studies that did not report outcome data, 14 conference abstracts or poster studies without available data, seven studies that were not randomized controlled trials, and three studies excluded for other reasons (different intervention methods) were removed. As a result, 11 studies were ultimately included in the meta-analysis.

### 2.3. Criteria for Inclusion and Exclusion of Data

This study examined the effects of RAS using wearable devices on the gait and balance of PD patients. Accordingly, the inclusion and exclusion criteria were defined as follows.

Inclusion Criteria

(1) Studies involving adult patients diagnosed with PD, regardless of the disease duration;

(2) Studies that applied RAS assisted by wearable devices;

(3) Studies in which the outcome measures were related to gait or balance;

(4) Studies presenting clear statistical results in the form of tables or text;

(5) Studies designed as randomized controlled trials (RCTs);

(6) Studies published in English.

Exclusion Criteria

(1) Studies conducted on patients with other neurological disorders or healthy individuals;

(2) Studies using feedback interventions other than RAS;

(3) Studies that used wearable devices only as assessment tools rather than as intervention tools;

(4) Studies whose outcome measures were unrelated to gait or balance;

(5) Abstracts, posters, or conference proceedings in which quantitative data were not provided;

(6) Studies that were not randomized controlled trials.

### 2.4. Data Extraction and Organization

The data from the 11 included studies were extracted and organized using a standardized data extraction form. The extracted items included the study title, author names, year of publication, total number of participants at the baseline (*n*), sex distribution, and the mean age and characteristics of the participants. The intervention frequency, intervention duration, and intervention setting were classified to organize the intervention protocols, and details of the rhythmic auditory stimulation techniques were recorded, including the type of music used, tempo, and specific characteristics. Information regarding wearable devices was also categorized into the device type, mode of feedback delivery, and device-specific features. Among the quantitative outcome data, all gait-related parameters provided by the wearable devices, such as gait speed, step length, and cadence, were extracted and organized. Similarly, all balance-related parameters provided by the wearable devices, including the BBS and the TUG, were also extracted and organized.

### 2.5. Data Analysis

Meta-analysis was conducted for this study using Review Manager 5.4 (RevMan 5.4). Mean differences (MD) were calculated when outcomes were measured using the same scale across studies. When outcomes were reported using different measurement scales, standardized mean differences (SMD) were calculated to allow comparisons across studies. A random-effects model was used to account for potential clinical and methodological heterogeneity among the included studies. To estimate the effect sizes, data on sample size (n), mean (m), and standard deviation (sd) were extracted from the experimental and control groups of each included study and analyzed. In studies where data were reported as medians, standard errors, interquartile ranges, or minimum–maximum values rather than the means and standard deviations, the values were converted into means and standard deviations to calculate the effect sizes. The level of statistical significance was set at *p* < 0.05.

The analyzed data are presented using forest plots, through which Z values, *p* values, and I2 values were provided to interpret statistical significance. The Z value is a test statistic that follows a standard normal distribution and is used to calculate the *p* value. A larger Z value indicates greater statistical significance, and a Z value of 1.96 corresponds to a significance level of 0.05. Therefore, when the Z value exceeds 1.96, the *p* value becomes less than 0.05, indicating a significant difference.

The I2 value represents the heterogeneity and indicates, as a percentage (%), the proportion of total variation across studies that is due to heterogeneity rather than chance. The classification is as follows:

(1) 0–25%: very low heterogeneity;

(2) 25–50%: moderate heterogeneity;

(3) 50–75%: high heterogeneity;

(4) ≥75%: very high heterogeneity.

Higher heterogeneity indicates greater differences in the results among the included studies. Differences in participant characteristics, intervention procedures, and assessment tools lead to greater heterogeneity, necessitating cautious interpretation of the results.

Sensitivity analysis was conducted to determine if any individual study exerted an excessive influence on the overall effect. Typically, a leave-one-out approach was used, in which studies were sequentially removed to examine the changes in effect size and heterogeneity. Results that did not change substantially were interpreted as indicating the stability of the findings. Accordingly, sensitivity analyses were performed for outcomes with high heterogeneity.

Subgroup analyses were conducted to compare effect sizes according to the participant characteristics, intervention methods, wearable device features, and assessment tools. The main variables for subgroup analyses included the frequency, type, and number of RAS sessions; the characteristics and types of wearable devices; and the types of outcome assessment tools used.

### 2.6. Assessment of Study Quality

(1) PEDro scale

The Physiotherapy Evidence Database (PEDro) scale was used to assess the methodological quality of the selected studies. This scale is applied to randomized controlled trials (RCTs) and is designed to evaluate the study quality. It consists of 11 items, each scored as 1 point if a criterion is met. The first item is not included in the total score, so the maximum possible score is 10. Each study was rated individually, and the average score across all included studies was calculated to determine the overall methodological quality of the selected studies for meta-analysis. The average scores were classified into three levels:

(1) 6–10 points: high methodological quality;

(2) 4–5 points: moderate methodological quality;

(3) ≤3 points: low methodological quality.

**Figure 2:**
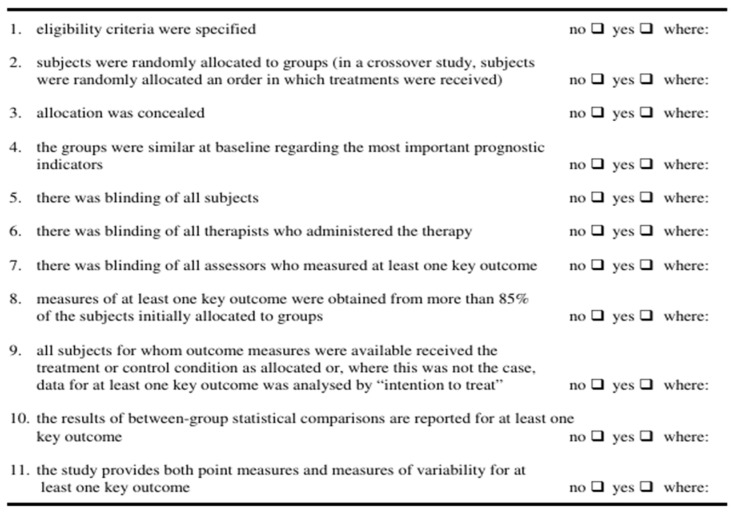
**PEDro** **item criteria used for methodological quality assessment.**

The detailed criteria of the PEDro scale are shown in Figure 2.

(2) ROB-2 (Risk of Bias 2.0)

The Cochrane Risk of Bias 2.0 (ROB-2) tool was used to assess the quality of the selected studies. This tool was designed to evaluate potential bias in randomized studies across the following five domains, with each domain classified as Low, Some Concerns, or High risk of bias:

(1) Randomization process;

(2) Deviations from intended interventions;

(3) Missing outcome data;

(4) Measurement of the outcome;

(5) Selection of the reported result.

**Figure 3:**
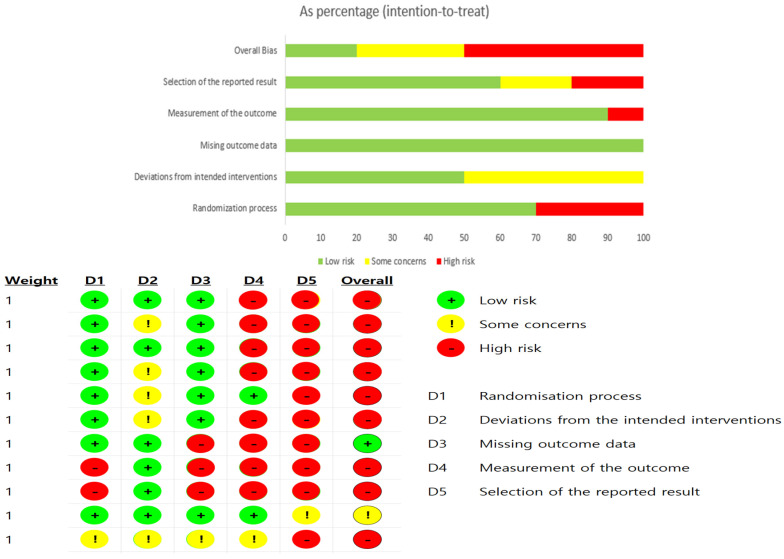
ROB 2.0.

Each domain consists of multiple signaling questions that allow for an objective assessment of methodological quality. For this study, the scoring was conducted using the Excel spreadsheet provided by Cochrane (Microsoft Excel), and the results were cross-checked and visualized. The outcomes of the ROB-2 assessment were presented as domain-specific proportion graphs and summary tables by study (Figure 3).

(3) GRADE (Grading of Recommendations Assessment, Development and Evaluation)

The GRADE (Grading of Recommendations Assessment, Development and Evaluation) approach was applied to assess the level of evidence from the included studies. GRADE is a tool used in meta-analyses to assess the certainty of evidence for each outcome, starting with randomized controlled trials at a high level of evidence and downgrading the certainty based on five assessment domains [39].

The GRADE assessment was conducted based on the following five domains:

(1) Risk of bias;

(2) Inconsistency;

(3) Indirectness;

(4) Imprecision;

(5) Publication bias.

These five domains were collectively considered to classify the certainty of evidence into four levels: high, moderate, low, and very low. The GRADE assessment results are presented in a Summary of Findings table.

## 3. Result

### 3.1. General Overview of Selected Studies

(1) General Characteristics of Studies

Table 1 lists the 11 studies finally included in this review. The 11 randomized controlled trials included targeted PD patients aged 60–70 years, and the study designs included parallel and crossover designs. The disease duration ranged from four to 15 years, and the Hoehn and Yahr stage was confirmed to be approximately stage 2 on average. Therefore, most participants were considered to have mild to moderate Parkinson’s disease.

The outcome variables measured included gait parameters, such as gait speed, step length, and cadence, as well as balance-related measures such as the BBS, TUG, and Mini-BESTest. In addition, there were outcome measures related to the upper extremities or trunk that indirectly affect balance and gait. In particular, several measures related to mental, psychological, and cognitive domains—such as the Falls Efficacy Scale–International (FES-I), Montreal Cognitive Assessment (MoCA), Parkinson’s Disease Questionnaire (PDQ), depression-related measures, and the Short Form (SF)—were also frequently observed, indicating that these factors may also influence gait and balance. The reported *p*-values of the individual studies for outcomes included in the meta-analysis are additionally presented in Supplementary Table S6.

(2) Protocol of Rhythmic Auditory Stimulation

Table 2 lists the RAS protocols applied in the 11 studies included in this review. In most studies, wearable devices equipped with inertial measurement units (IMUs) were attached between the knee and the heel. Specifically, Most studies used IMU-based wearable devices attached below the knee and heel, although a few studies used head- or wrist-worn devices. Various types of audio output equipment were used, including earphones, headphones, and bone-conduction headphones.

In many studies, the participants’ individual rhythms were measured before the start of training, either over a specified period of time or across a set number of steps. Training typically began at the participant’s current rhythm or speed, or at a slightly faster pace, and the rhythm or speed was adjusted based on whether predefined conditions were met. In addition, rather than using a simple metronome solely to provide real-time feedback and record participant data, many studies used smartphone applications or computer-based programs to enhance the objectivity of the assessment and the quality of training. Another notable feature was the setting of intervention delivery. Many studies did not limit their application to hospital settings because of the characteristics of RAS, but instead implemented interventions in home and community environments.

**Figure 4:**
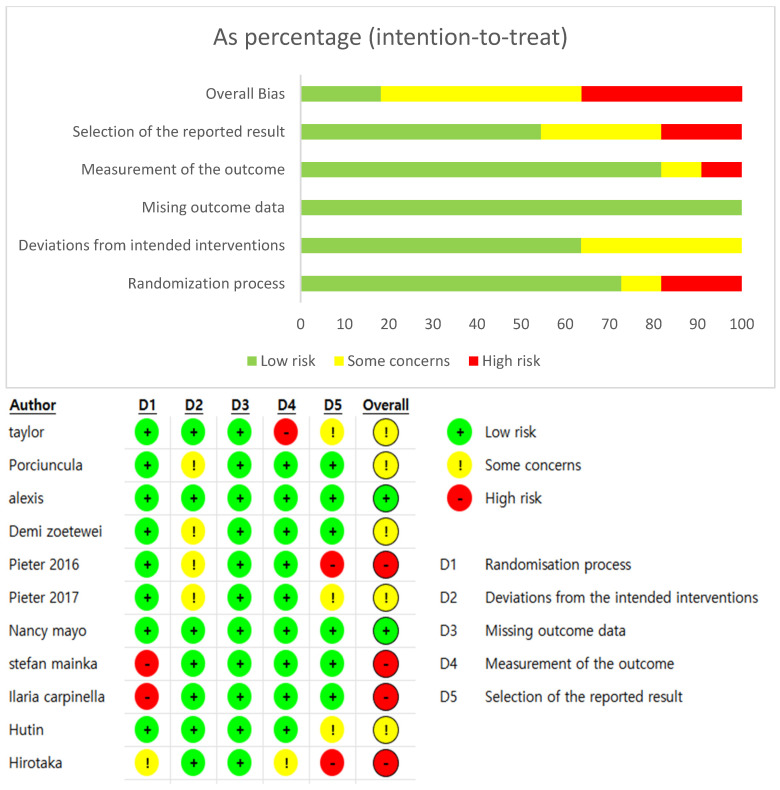
ROB-2 (Risk of Bias 2.0).

The ROB-2 tool comprises multiple domains, enabling an objective evaluation of the methodological quality. For this study, scores were recorded using an Excel sheet provided by Cochrane (Microsoft Excel), and cross-checking was performed to enable visualization. The results of the ROB-2 assessment are presented as domain-level proportion graphs and a study-level summary table (Figure 4).

### 3.2. Quality and Risk of Bias Assessment of Selected Studies

(1) Methodological Quality Assessment (PEDro Scale)

Table 3 presents the methodological quality assessment of the 11 included studies using the PEDro Scale. The mean PEDro score was 7.27, indicating a moderate level of methodological quality. Most studies met the criteria for random allocation, baseline comparability, data adequacy, and between-group comparisons. On the other hand, blinding of therapists, assessors, and participants was generally difficult to achieve because of the inherent characteristics of the training interventions.

(2) Summary and Distribution of Risk of Bias (ROB-2)

The risk of bias of the 11 included studies was assessed using the ROB 2.0 tool (Figure 4). High risk or some concerns were identified in the domains of the randomization process and selective reporting of outcome data. In addition, the blinding procedures were unclear in several studies because of the nature of the training interventions, resulting in observations of high risk or some concerns. Regarding the objectivity and accuracy of outcome data and assessment tools, most studies showed a low risk of bias.

### 3.3. Meta-Analysis

(1) Meta-Analysis and Publication Bias of Gait Speed

**Figure 5:**
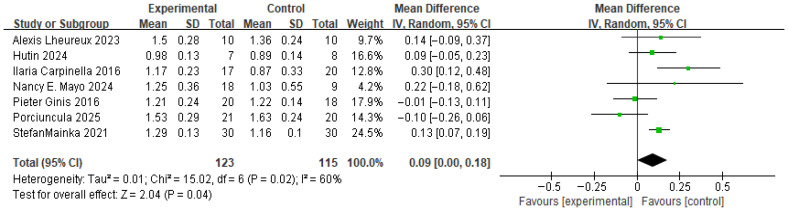
Forest plot of Gait speed. Green squares indicate the effect estimates of individual studies, and the black diamond indicates the pooled overall effect.

Figure 5 presents the results for gait speed. Based on a forest plot derived from seven studies, the experimental group that received wearable device-based RAS showed a statistically significant improvement compared with the control group (MD = 0.09, *p* < 0.04). The between-study heterogeneity was 60%, indicating moderate heterogeneity. Accordingly, a sensitivity analysis was performed. When each study was sequentially excluded, the pooled effect size consistently remained in a positive direction, and no substantial changes in heterogeneity were observed (range: 0.06–0.12). These findings indicate that no single study disproportionately influenced the overall results.

**Figure 6:**
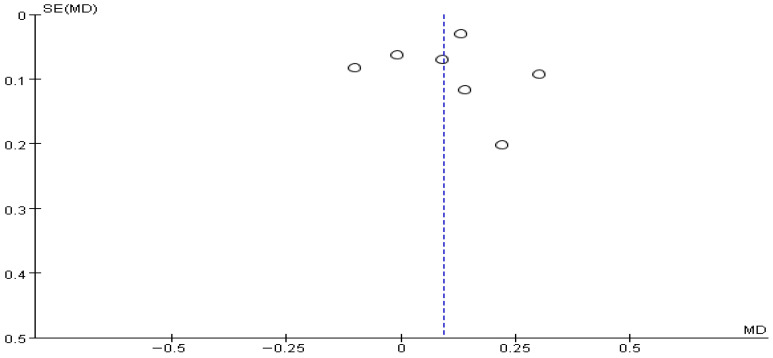
Funnel plot of Gait speed. The dotted vertical line represents the overall effect estimate.

The funnel plot for gait speed showed a generally symmetrical distribution, and a visual inspection did not reveal any apparent asymmetry or directional bias, suggesting no evident publication bias (Figure 6).

(2) Meta-Analysis and Publication Bias of Cadence

**Figure 7:**
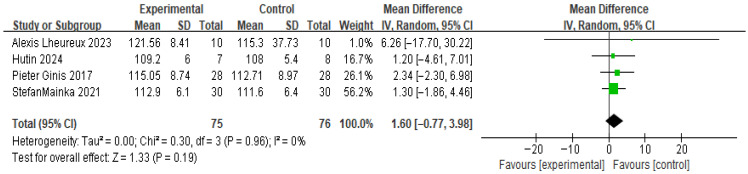
Forest plot of Gait Cadence. Green squares indicate the effect estimates of individual studies, and the black diamond indicates the pooled overall effect.

Figure 7 shows the results for gait cadence. Based on the forest plot derived from four studies, no significant difference was observed between the experimental group that received RAS and the control group. The effect size was 1.06, with a *p* value of 0.19, indicating no significant difference. The between-study heterogeneity was 0%, suggesting consistent directional effects across studies.

**Figure 8:**
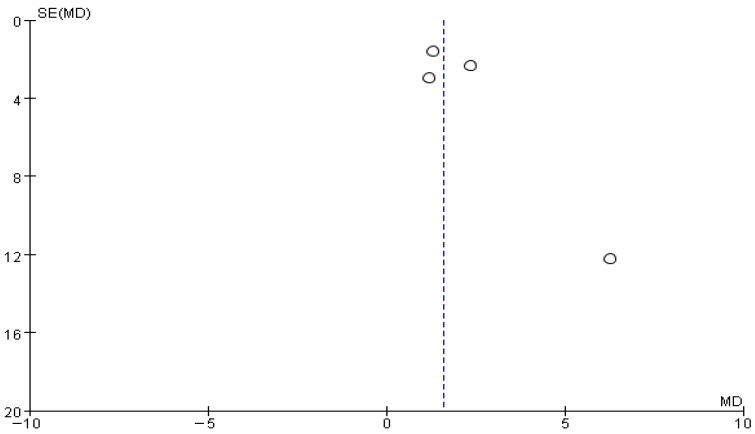
Funnel plot of Gait Cadence. The dotted vertical line represents the overall effect estimate.

The funnel plot for gait cadence showed partial asymmetry. Most studies were distributed near the baseline, but one study was located in the lower-right region of the plot (Figure 8).

(3) Meta-Analysis and Publication Bias of Gait Spatial

**Figure 9:**
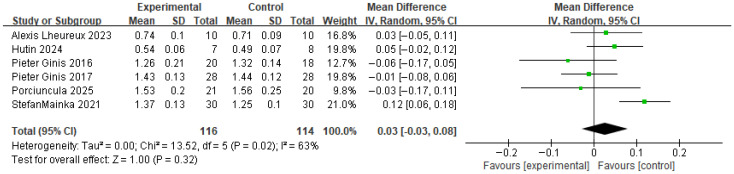
Forest plot of Gait Spatial. Green squares indicate the effect estimates of individual studies, and the black diamond indicates the pooled overall effect.

Figure 9 presents the results for gait spatial parameters. This variable integrates the step length and the stride length. Based on the forest plot derived from six studies, no significant difference was observed between the experimental group that received RAS and the control group. The effect size was 0.03, with a *p* value of 0.32, indicating no significant difference. The between-study heterogeneity was 63%, indicating moderate heterogeneity.

**Figure 10:**
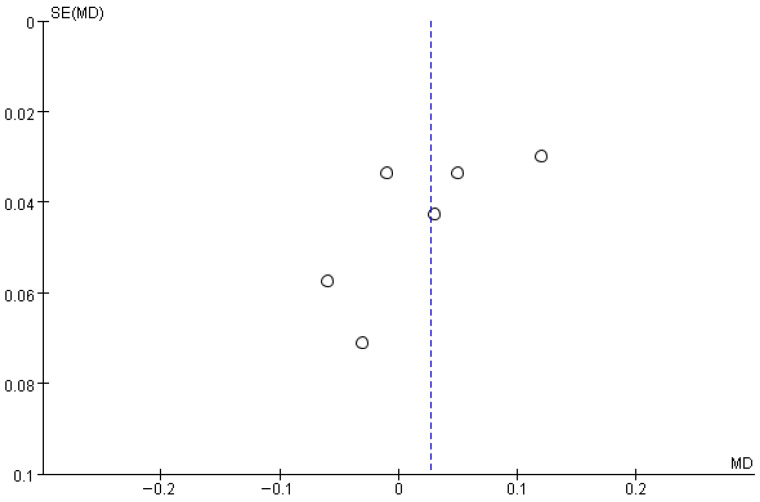
Funnel plot of Gait Spatial. The dotted vertical line represents the overall effect estimate.

The funnel plot for the gait spatial parameters showed a generally symmetrical distribution, and a visual inspection did not reveal any apparent asymmetry or directional bias, suggesting no evident publication bias (Figure 10).

(4) Meta-Analysis and Publication Bias of Gait Variability

**Figure 11:**
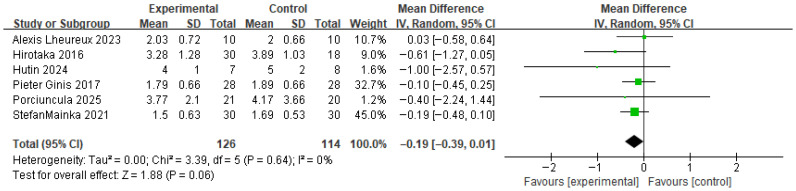
Forest plot of Gait variability. Green squares indicate the effect estimates of individual studies, and the black diamond indicates the pooled overall effect.

Figure 11 shows the results for gait variability (step time variability). Based on the forest plot derived from six studies, no significant difference was observed between the experimental group that received RAS and the control group. The effect size was −0.19, with a *p* value of 0.06, indicating no significant difference. The between-study heterogeneity was 0%, indicating consistent directional effects across studies.

**Figure 12:**
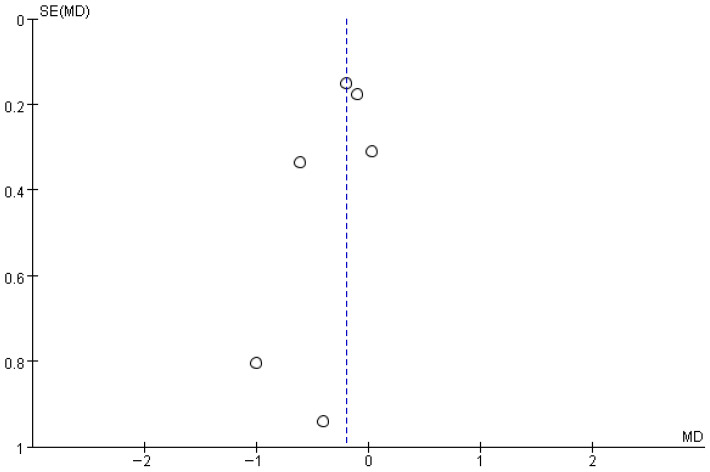
Funnel plot of Gait variability. The dotted vertical line represents the overall effect estimate.

The funnel plot for gait variability showed a generally symmetrical distribution, and a visual inspection did not reveal any apparent asymmetry or directional bias (Figure 12).

(5) Meta-Analysis and Publication Bias of Balance Ability

**Figure 13:**
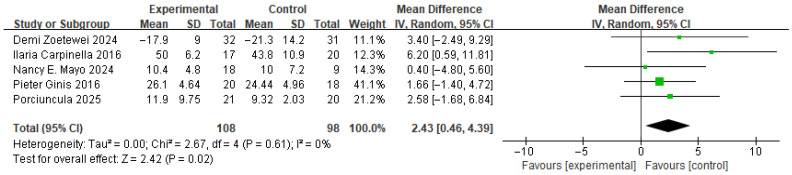
Forest plot of Balance ability. Green squares indicate the effect estimates of individual studies, and the black diamond indicates the pooled overall effect.

Figure 13 shows the results for balance ability. This variable integrated the BBS, Mini-BESTest, TUG, and Four Square Step Test (FSST). tTherefore, standardized mean difference (SMD) were used to synthesize the results. For TUG, lower values indicate improvement. Therefore, negative (–) signs were applied. Based on the forest plot derived from five studies, a significant difference was observed between the experimental group that received RAS and the control group. The effect size was 2.43, indicating a moderate effect, and the *p* value was 0.02, indicating a significant difference. The between-study heterogeneity was 0%, indicating consistent directional effects across studies.

**Figure 14:**
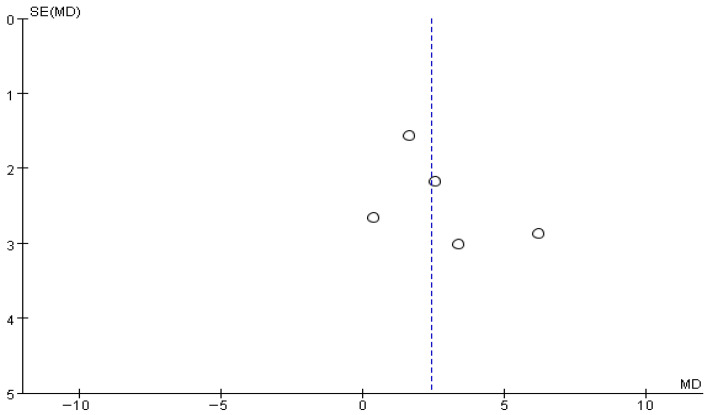
Funnel plot of Balance ability. The dotted vertical line represents the overall effect estimate.

The funnel plot for balance ability showed a generally symmetrical distribution, and a visual inspection did not reveal any apparent asymmetry or directional bias (Figure 14).

(6) Meta-analysis and publication bias for FOG-Q

**Figure 15:**
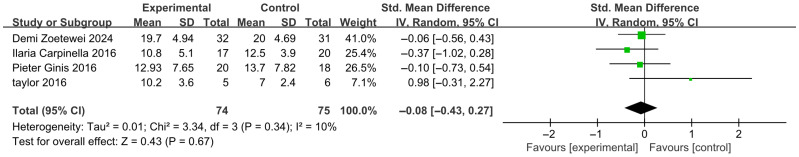
Forest plot of FOG-Q (Freezing of Gait Questionnaire). Green squares indicate the effect estimates of individual studies, and the black diamond indicates the pooled overall effect.

Figure 15 shows the results for the Freezing of Gait Questionnaire (FOG-Q). Based on the forest plot derived from four studies, no significant difference in effect was observed between the experimental group that received RAS and the control group. The effect size was −0.08, with a *p* value of 0.67, indicating no significant difference. The between-study heterogeneity was 10%, showing generally consistent directional effects across studies.

**Figure 16:**
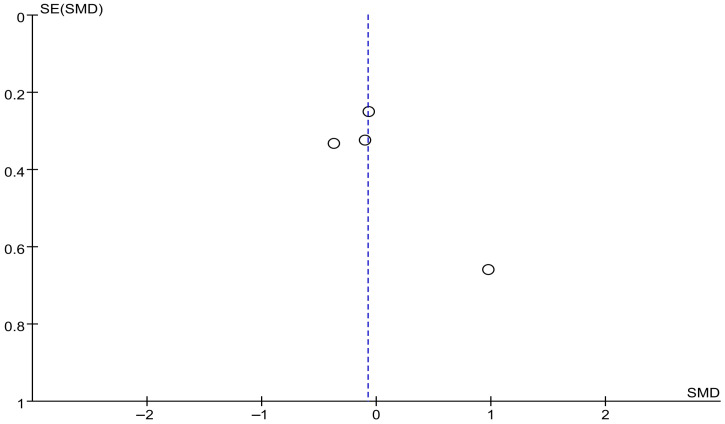
Funnel plot of FOG-Q. The dotted vertical line represents the overall effect estimate.

The funnel plot for FOG-Q showed partial asymmetry. Most studies were distributed near the reference line, but one study was located relatively in the lower right region of the plot (Figure 16).

(7) Meta-analysis and publication bias for UPDRS-III

**Figure 17:**
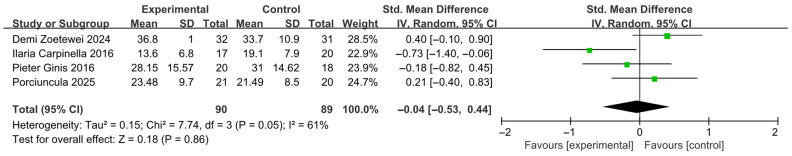
Forest plot of UPDRS-III (Unified Parkinson’s Disease Rating Scale-III, Motor Function). Green squares indicate the effect estimates of individual studies, and the black diamond indicates the pooled overall effect.

Figure 17 presents the results for the Unified PD Rating Scale Part III (UPDRS-III). Based on the forest plot derived from four studies, no significant difference in effect was observed between the experimental group that received RAS and the control group. The effect size was −0.04, with a *p* value of 0.86, indicating no significant difference. The between-study heterogeneity was 61%, indicating moderate heterogeneity, and a relatively large variability among individual studies was observed. Accordingly, a sensitivity analysis was conducted. When the study by Ilaria was excluded, the heterogeneity decreased to 0%, suggesting that this study may have contributed substantially to the observed heterogeneity. Despite this, the pooled effect size remained within the range of −0.32 to 0.12, indicating that no single study had a decisive influence on the overall results.

**Figure 18:**
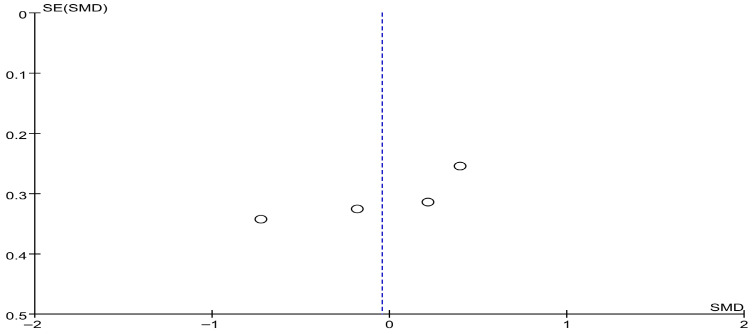
Funnel plot of UPDRS-III (Unified Parkinson’s Disease Rating Scale-III, Motor Function). The dotted vertical line represents the overall effect estimate.

The funnel plot for UPDRS-III showed a generally symmetrical distribution, and a visual inspection did not reveal any apparent asymmetry or directional bias (Figure 18).

### 3.4. GRADE (Grading of Recommendations Assessment, Development and Evaluation)

The certainty of evidence for each outcome variable was evaluated using the GRADE approach (Table 4). Moderate certainty of evidence was identified for gait speed and balance outcomes. Cadence, gait spatial parameters, gait variability, and the FOG-Q were rated as having a low certainty of evidence. The certainty of evidence for UPDRS-III was classified as very low.

## 4. Discussion

This study is a systematic review and meta-analysis that examined the effects of RAS using wearable devices on the gait and balance in PD patients. The results revealed statistically significant improvements in gait speed and balance ability, whereas no significant differences were observed in cadence, spatial parameters, gait variability, FOG-Q, or UPDRS-III. An examination of heterogeneity (I2) and publication bias (funnel plots) indicated that most outcomes showed low heterogeneity and symmetrical distributions, suggesting minimal publication bias. Regarding the methodological quality, the mean PEDro score was 7.27, indicating high study quality. According to the ROB-2 assessment, several studies were rated as high risk in the randomization domain, whereas most studies showed some concerns or low risk. Therefore, the findings should be interpreted with caution despite the significant improvements observed in gait speed and balance. The systematic review showed that RAS combined with wearable devices was applied to PD patients aged 60 years and older, using devices attached to various body locations. In addition to the gait and balance variables, the mental, psychological, and cognitive outcomes were also frequently assessed. An examination of the intervention protocols showed that most wearable devices incorporated inertial measurement units (IMUs) mounted on areas, such as the knee, wrist, ankle, or heel, to collect real-time patient data. These technologies include sensor-based wearable systems, smartphone applications, and IMU-based motion detection devices that provide real-time auditory feedback during walking training. The data were continuously collected using applications or programs operating on smartphones, computers, or tablets. The collected information was used to provide feedback corresponding to the predicted cadence and deliver corrective feedback when the preset cadence was not maintained, enabling the effective training of movement rhythm. Feedback modalities included auditory signals, vibration, laser cues, visual markers, and voice guidance, but most RAS interventions primarily provided metronome sounds. A notable feature of these interventions was that training was conducted predominantly in home settings [32,40] or community-based environments rather than in hospitals [29,41]. From a clinical perspective, many of these wearable systems are relatively low-cost because they can be implemented using commercially available sensors and smartphone-based applications. This accessibility may facilitate the practical adoption of RAS in rehabilitation settings.

The results for the gait speed in this study showed significant improvement, along with low heterogeneity and no apparent publication bias. This finding suggests that wearable device-based RAS is beneficial for improving the gait speed. RAS generates a cadence through the repetition of regular sounds. This cadence initially matches the participant’s baseline walking rhythm measured before training. Nevertheless, as training progresses, the cadence is gradually increased by approximately 5–10%, and participants are instructed to walk in synchrony with the adjusted rhythm [42]. Because PD patients exhibit reduced intrinsic timing generation due to a basal ganglia dysfunction, the RAS compensates for timing deficits by providing regular and repetitive rhythmic cues that facilitate stable movement rhythms. As patients become more accustomed to the training, gait speed naturally increases, which likely explains the observed results [43]. These findings are consistent with previous meta-analyses reporting that RAS significantly increases the gait speed [44]. Previous meta-analyses have also reported that improvements were primarily observed in gait speed, while changes in other gait parameters were relatively limited. Because gait speed reflects the combined influence of cadence and step/stride length, small non-significant changes in these individual components may collectively contribute to a significant improvement in overall gait speed.

In this study, the gait cadence results showed no significant difference, and low heterogeneity and no evident publication bias were observed. This finding may be explained by the characteristic nature of cadence, which reflects the maintenance of rhythm rather than speed among gait components, resulting in limited observable change. In PD, the patients experience difficulty sustaining continuous walking and often exhibit a shortened step length, leading to impairments in cadence [45]. On the other hand, RAS does not focus primarily on increasing the cadence itself, but rather on restoring the original rhythm when walking is interrupted or when steps become abnormally shortened [42]. Previous studies have also reported that the magnitude of improvement across various gait patterns is limited. Although rhythmic cues have an immediate effect on the timing of the gait rhythm, longer-term kinematic relearning is required to induce changes in spatial variables such as the step length or cadence [42].

In this study, no significant differences were found for the gait spatial parameters or gait variability. Although some studies have reported improvements in the spatial gait variables, these outcomes were achieved through long-term training conducted in controlled environments under the supervision of specialized personnel [46]. In contrast, many studies included in this meta-analysis regarding the devices provided to participants allowed them to train independently in home or community settings without continuous therapist supervision. Under such conditions, insufficient intervention duration or limited repetitive practice may fail to produce observable improvements in gait patterns [40]. As noted earlier, the spatial gait variables require longer-term kinematic relearning [42]. Furthermore, several studies included in this meta-analysis used relatively short intervention periods of four or six weeks and a limited number of sessions, which may have influenced the results. The gait patterns are determined by complex interactions involving neuromuscular coordination, trunk rotation, and segmental joint actions. Hence, they require a relatively longer learning period than the gait speed [26]. Therefore, it is likely that short-term RAS interventions were insufficient to produce meaningful spatial corrections in step length, cadence, or gait variability. Another possible explanation for the limited effects observed in some gait parameters may be related to cognitive limitations and dual-task difficulties in individuals with Parkinson’s disease. Because patients are required to process auditory cues while simultaneously performing walking tasks, the cognitive load associated with dual-task processing may reduce the effectiveness of RAS in some cases.

Another contributing factor may be that only auditory sensory feedback was provided. Although self-recognition and problem-solving by the participant are important for correcting the motor performance, more effective outcomes may be achieved when auditory feedback is combined with visual and somatosensory information, along with immediate and active guidance from a therapist [47]. Even if wearable devices provided consistent auditory feedback, the absence of a therapist who could observe the overall gait process from a broader perspective represents a limitation. Therefore, RAS appears to have limited effects on fine-grained gait pattern control, and similar outcomes are observed, even when wearable devices are used.

These results regarding the balance ability revealed a significant difference, with low heterogeneity and low publication bias. These findings suggest that RAS has a positive effect on the gait speed and various balance-related outcome measures. Balance is achieved through the integration of visual, vestibular, and somatosensory information [13,48], and the ability to integrate sensory information and regulate posture is critical for performance in various balance tasks [49]. In PD patients, impairments in postural responses, weight shifting, and responses to perturbations lead to difficulties in sensory integration and postural control [15]. RAS repeatedly presents salient auditory stimuli and requires the participants to perform movements in response to these repeated sounds. This approach facilitates the initiation and continuation of movement by embedding movement within a rhythm [42]. The entrainment characteristic of rhythm, which produces predictable movement patterns, continuously promotes feedforward control and feedback of movement. The transfer of this training may help improve balance ability and motor learning when the evaluation tasks are similar to the trained movements. In this study, the balance outcomes were analyzed by integrating the BBS, Mini-BESTest, TUG, and FSST. These assessments evaluate the balance ability by scoring the performance or measuring the time across various functional tasks [50,51,52]. Common components among these tasks include movements such as standing up from a seated position, walking, and turning. Because these movements are also fundamental elements of gait, they can be sufficiently incorporated into RAS training procedures. As training progresses, the participants may also perform a variety of functional gait-related tasks, many of which correspond to the more challenging items included in balance assessments. Therefore, with longer training durations, the participants are likely to have engaged in a broader range of balance-related exercises. In addition, RAS using wearable devices provides feedback based on real-time data collection, enabling the system to notify the participants of errors when the postural changes exceed predefined thresholds [29]. These features are believed to have contributed to improvements in balance ability.

The FOG-Q results did not show a significant difference, and low heterogeneity and largely symmetrical publication bias were observed, except for one study. The FOG-Q is an assessment tool consisting of six items designed to evaluate the freezing of gait in PD patients, with higher scores indicating more severe freezing [53]. Although RAS has been reported to reduce freezing of gait in PD patients [54], no significant difference was observed in this study. This result may be because most of the included studies used short-term intervention programs of four to six weeks with a limited number of treatment sessions. Motor automaticity requires sufficient repetitive stimulation [42], and the short duration and low frequency of training in the included studies may not have met this requirement. In addition, factors that facilitate motor automaticity include immediate corrective instructions from a therapist when rhythmic errors occur during training [26]. Nevertheless, immediate and active therapist involvement was limited because the wearable device-based RAS interventions in the included studies were conducted primarily in home or community settings, making it difficult to provide correction or guidance for the freezing of gait.

The UPDRS-III results did not show a significant difference, and moderate heterogeneity was observed. Nevertheless, the results can be considered reliable because symmetrical publication bias was identified. The UPDRS-III is an assessment tool designed to evaluate the motor function in PD patients by scoring a wide range of functional items [55], with higher scores indicating greater impairment. The findings of this meta-analysis indicate limitations in producing changes in the overall motor function, bradykinesia, postural instability, and other complex motor symptoms within short intervention periods and with a limited number of training sessions, similar to the outcomes observed for other variables. The recovery of motor automaticity and motor control requires long-term repetitive training. Therefore, it is also considered necessary to integrate multisensory training approaches [26]. Furthermore, although the UPDRS-III includes items related to gait, postural stability, and balance, it also assesses speech, facial expression, finger movements, and upper limb movements. In addition, difficult questions and cultural differences may introduce variability in scoring [56]. When these factors are considered, it is unlikely that real-time auditory feedback from wearable device-based RAS alone would sufficiently improve the UPDRS-III scores. Therefore, to improve the UPDRS-III outcomes, RAS should be combined with other therapeutic interventions, and more detailed and comprehensive assessments should be conducted to achieve overall functional improvement.

As described above, RAS generates a tempo through the repetition of regular sounds. It causes entrainment between the tempo and movement, sustains movement to induce automatization. In the later stages of training, it facilitates translation, in which the target movements are performed without auditory cues, enabling their application in the individual’s real-life environment. In this context, RAS is fundamentally based on entrainment to external auditory cues. Thus, the auditory rhythm primarily functions as a feedforward cue that facilitates synchronization between movement and tempo, rather than as feedback alone. Wearable devices may additionally support this process through real-time monitoring and supplementary feedback [42]. Owing to these characteristics, therapists can provide training-based guidance for training that involves repetitive movement components during rehabilitation. This allows for diverse approaches such as group exercise and equipment-based training, reduces the physical burden on individual therapists, and adds an element of interest to otherwise simple repetition, enabling sustained training [26].

The wearable devices used in this study refer not to digitized assessment tools, but to wearable devices used as training aids. In other words, they function as rehabilitation support devices through real-time feedback. Their significance lies in enabling more objective and efficient patient-centered self-training by moving away from a rehabilitation structure that requires constant therapist presence and from environments in which rehabilitation is confined to hospitals [57]. Although the magnitude of improvement may be comparable to that of conventional free-field RAS, wearable RAS may provide added value by supporting more personalized, continuous, and ecologically relevant gait training beyond supervised clinical settings. An examination of the studies included in this meta-analysis showed that most used IMU sensors to detect the patients’ gait patterns in real time and to provide auditory, tactile, and visual feedback corresponding to the detected signals. Considering the ongoing technological advances, future developments are expected to expand the predictive capacity of data, enabling the provision of more diverse bodily information and a broader range of sensory feedback [58].

Reviewing studies that delivered RAS using wearable devices has several advantages. First, mobility and accessibility are improved because wearable devices can be applied in hospitals as well as in home and community settings. This approach is considered an effective method for the continuous health management of patients after discharge. Second, wearable devices serve as rehabilitation tools that enable individualized training. Rather than merely adjusting the difficulty levels or providing monotonous exercises, device algorithms analyze the individual gait patterns and adjust training difficulty in real time. In addition, the ability to select from various types of music as training progresses may contribute to sustained engagement. Third, quantitative data collection and monitoring are possible. The data obtained through wearable devices enable an objective presentation of patient information, supporting remote rehabilitation alongside research applications. These advantages suggest that wearable device-based RAS can facilitate continuous rehabilitation for patients while providing therapists with the opportunities to manage care beyond hospital settings, and further development in this field is anticipated. Although significant improvements in gait speed and balance were observed, the magnitude of these effects should be interpreted with caution. The overall effect size was moderate, suggesting that while wearable device-based RAS may provide benefits for individuals with Parkinson’s disease, the clinical impact may vary depending on intervention protocols, device types, and patient characteristics.

Several limitations of this study should be acknowledged. First, the number of included studies was relatively small (11 studies), limiting the generalizability of the findings. Second, training procedures were not standardized, and the intervention duration, frequency, and types of feedback varied across studies. Establishing minimal standards may facilitate broader adoption, and this issue warrants further investigation. Third, some wearable cueing approaches may mainly affect step frequency rather than step length, which may limit functional improvement and raise safety concerns in Parkinson’s disease. Finally, the absence of long-term follow-up studies made it difficult to evaluate the persistence of intervention effects. Future meta-analyses may address these limitations as research in this area continues to develop. In addition, although significant improvements were observed in gait speed and balance, no significant effects were found in other gait parameters or freezing of gait, which should be considered when interpreting the results. Although the effect sizes were modest, improvements in gait speed and balance may still be clinically relevant for functional mobility in patients with Parkinson’s disease.

## 5. Conclusions

This study evaluated the effects of wearable device-based RAS on the gait and balance abilities of PD patients through a systematic review and meta-analysis. The results revealed improvements in gait speed and balance ability, which may be attributed to the key characteristics of this intervention, including anticipatory control and feedback provided by auditory stimulation, the feasibility of continuous repetitive training, and movement correction based on real-time information. Nevertheless, effects were not observed for other gait parameters, freezing of gait, or outcome measures assessing the broader motor functions.

These findings suggest that additional training or evaluation of long-term effects may be necessary. Therefore, this intervention may be used as a sustainable rehabilitation tool for PD patients, extending beyond the short-term, hospital-based treatment to home- and community-based settings. Therefore, future research should focus on standardizing training protocols and examining long-term interventions to verify the sustained effects, with the ultimate goal of developing clinical rehabilitation guidelines based on wearable device-assisted training.

## Figures and Tables

**Table 1:** Characteristics of Included Studies.

Author	Study Design	Control Condition	Subject (EG/CG)	Age (Mean ± SD)	Sex (M/F)	Disease Duration (Year, Mean ± SD)	UPDRS-III (Mean ± SD)	H & Y (Mean ± SD)	Primary Outcome	Secondary Outcome
Taylor	Parallel	Podcast playback	5/6	70.8 ± 5.6 69.0 ± 5.7	5/0 4/2	15.4 ± 5.4 11.2 ± 5	18.2 ± 6.4 20.3 ± 4.7	2.5 ± 0.5 2.7 ± 0.41	FES-I, FOG-Q, MoCA, DT cognitive	
Porciuncula	Parallel	No-cue walking	21/20	66.95 ± 8.84 60.65 ± 9.13	11/10 10/10	X	20.71 ± 4.68 22.75 ± 10.6	2.1 ± 0.6 2.0 ± 0.5	Daily moderate-intensity walking minutes, Daily step counts, STV	UPDRS-III, MINI-BESTest, Speed, 10MWT, 6MWT, FSST, PDQ-39, Depression Scale
Alexis	Repeated	No-cue walking	10	68.3 ± 9.3	6/4	5.6 ± 3.2	44.3 ± 17.8	2	Gait speed, Cadence, Stride, Mean Stride duration, STV	
Demi	Parallel	Step-count feedback	32/31	67.7 ± 7.97 68.7 ± 7.31	21/11 23/8	11.3 ± 6.80 11.8 ± 5.76	36.5 ± 10.8 34.8 ± 11.6	X	%TF, Number of FOG, Duration FOG	FOG-Q, TUG, 4MWT, MINI-BEST, UPDRS-III
Pieter 2016	Parallel	No-cue walking	20/18	67.30 ± 8.13 66.11 ± 8.07	17/3 13/5	10.65 ± 5.39 11.67 ± 7.63	28.35 ± 14.77 33.77 ± 14.36	2.2 ± 0.5 2.1 ± 0.4	Speed, Stride, STV, MINI-BEST, FES-I, FSST, 2MWT, UPDRS-III, FOG-Q, Cognition, SF-36	
Pieter 2017	Crossover	No-cue walking	28	62.04 ± 6.91	23/5	10.57 ± 6.71	34.57 ± 14.37	2.0 ± 0.5	Cadence, Stride length, Fatigue	Double Support time, Arm ROM STV
Nancy	Parallel	Verbal instruction	18/9	70.2 ± 8.5 70.7 ± 8.8	13/5 6/3	X	X	2.5 ± 0.5 2.5 ± 0.5	6MWT, STS, PDQ, VAS, QOL	Gait Parameter
Stefan Mainka	Repeated	Normal walking	30	62.1 ± 7.0	18/12	4.6 ± 3.8	21.1 ± 8.8	2.0 ± 0.6	Arm ROM, Cadence, Speed, Stride, STV, Sternum rotation	
Ilaria	Parallel	No-cue walking	17/20	73.0 ± 7.1 75.6 ± 8.2	14/3 9/11	7.5 ± 3.2 10.3 ± 5.7	16.6 ± 6.8 22.3 ± 7.3	2.7 ± 0.7 2.9 ± 0.5	BBS, 10MWT	UPDRS, TUG, ABC, FOG-Q, PDQ-39, COP
Hutin	Parallel	Metronome-based RAS	7/8	67.3 ± 20.2 70.3 ± 7.1	5/2 2/6	12.7 ± 3.0 11.7 ± 5.9	15.3 ± 6.4 29.3 ± 11.3	2~3	Speed, Step length, Cadence, STV	
Hirotaka	Repeated	No-cue walking	30/18	74.87 ± 7.10	14/16	6.05 ± 4.21	22.66 ± 7.35	2.77 ± 0.45	Stride interval, STV	

Mean ± SD: Mean, Standard Deviation; UPDRS-III: Unified Parkinson’s Disease Rating Scale-III (Motor Examination); H & Y: Hoehn and Yahr Scale; FES-I: Falls Efficacy Scale International; FOG-Q: Freezing of Gait Questionnaire; MoCA: Montreal Cognitive Assessment; DT: Dual Task; MINI-BESTest: Mini Balance Evaluation; 10MWT: 10 m walk test; 6MWT: 6 min walk test; PDQ-39: Parkinson’s Disease Questionnaire-39; SF-36: Short Form 36 Health Survey; FSST: Five time Sit-to-Stand; STV: Stride Length Variability (CV%); TF: time frozen; 4MWT: 4 m walk test; STS: Sit to stand; VAS: Visual Analog Scale; QOL: Quality of Life Survey; BBS: Berg Balance Scale; TUG: Timed up and go test; ABC: Activities-specific Balance Confidence Scale; EG: Experimental group; CG: Control group.

**Table 2:** Structure of the Wearable rhythmic auditory stimulation protocols.

Author	Device	Feature	Site	Duration	Session	Feedback	Place
Taylor	Ambulosono Platform (Music Player + IMU sensor + Earphone)	Plays music when leg elevation exceeds threshold	Knee	4 weeks ≥3 times	10~ 20 min	Music	Home
Porciuncula	Music based Digital RAS system (MR-005, Medrhythms) IMU Shoes + Bone Conduction Auditory Device + Touch Screen	Tempo set after 20 steps → automatically adjusted by ±5%	Shoes	6 weeks ≥5 times	30 min	Music	Community
Alexis	Headphone + Smartphone +App (soundbrenner)	Tempo set after 256 steps → adjusted by ±10%	Head	1 session	5~7 min	Metronome	Hospital
Demi	DeFOG system (IMU shoes + Smartphone + Earphone)		Shoes	4 weeks ≥3 times		Music	Home
Pieter 2016	CuPid System (IMU shoes + Smartphone + Earphone) App (ABF gait + FOG Cue)	Tempo set after 1 min ABF: positive feedback if target cadence is maintained, corrective feedback if not maintained; FOG: cue provided when FOG detected	Ankle	6 weeks ≥3 times	30 min	Metronome, Voice, Color change	Home
Pieter 2017	IntCue/ConCue/IntFB (Headphone + IMU shoes + Computer)	Tempo set after 1 min ConCue: adaptive metronome provided; IntCue: metronome when cadence not maintained; IntFB: voice guidance when cadence not maintained	Dorsum	6 weeks ≥3 times	30 min	Metronome, Voice	
Nancy	Heel2Toe Program Earphone + Smartphone + App (Heel2Toe)	Feedback automatically set after heel-strike detection	Heels	12 weeks ≥5 times	≥5 min	Metronome	home
Stefan Mainka	Curaswing Earphone + Wrist IMU + Smartphone + App (Curaswing)	Tempo set after 1 min walking → adjusted by ±5%	Wrist	1 session	20~ 25 min	Music	
Ilaria	Game Pad Sys IMU (Sternum, Sacrum, Thigh, Calf) + Computer	Warning sound when movement deviates from the normal range (angle, speed)	Whole	7 weeks ≥3 times	45 min	Metronome, Visual	
Hutin	Gait Tutor system IMU (foot, sternum) + earphone + app	Voice guidance provided when gait speed falls below the preset target (110% of baseline)	Ankle, Trunk	1 session	20 min	Metronome, Voice	
Hirotaka	Walk-Mate system (Headphone + PC + pressure sensor)	Rhythm calculated by measuring foot-contact timing using pressure sensors	Shoe	1 session		Metronome, Voice	

IMU: Inertial Measurement Unit; App: application; FOG: Freezing of Gait.

**Table 3:** PEDro Scale.

Study	1	2	3	4	5	6	7	8	9	10	11	Total
Taylor	O	X	O	O	O	X	X	O	X	O	O	7
Porciuncula	O	O	O	O	X	X	O	O	O	O	O	9
Alexis	O	O	X	O	X	X	O	O	O	O	O	8
Demi	O	O	X	O	X	X	X	O	O	O	O	7
Pieter 2016	O	O	O	O	X	X	X	O	O	O	O	8
Pieter 2017	O	O	X	O	X	X	X	O	O	O	O	7
Nancy	O	O	X	O	O	X	X	X	O	O	O	7
Stefan Mainka	O	O	X	O	X	X	X	O	O	O	O	7
Ilaria	O	O	X	X	X	X	O	O	X	O	O	6
Hutin	O	O	O	O	O	X	O	O	X	O	O	9
Hirotaka	O	X	X	O	X	X	X	X	O	O	O	5
Total 7.27												

**Table 4:** GRADE.

Outcome	No. of Studies	Effect Estimate (MD, 95% CI)	Risk of Bias	Inconsistency	Indirectness	Imprecision	Publication Bias	Overall Certainty
Gait speed	7 RCTs	MD = 0.09 (0.00~0.18)	Serious	Not Serious	Not Serious	Not Serious	Undetected	●●●◯ Moderate
Cadence	6 RCTs	MD = 1.60 (−0.77~3.98)	Serious	Not Serious	Not Serious	Serious	Suspected	●●◯◯ Low
Gait Spatial parameter	6 RCTs	MD = 0.03 (−0.03~0.08)	Serious	Serious	Not Serious	Serious	Undetected	●●◯◯ Low
Gait variability	6 RCTs	MD = −0.19 (−0.39~0.01)	Serious	Not Serious	Not Serious	Serious	Undetected	●●◯◯ Low
Balance	5 RCTs	MD = 2.43 (0.46~4.39)	Not Serious	Not Serious	Serious	Not Serious	Undetected	●●●◯ Moderate
FOG-Q	4 RCTs	SMD = −0.08 (−0.43~0.27)	Serious	Not Serious	Not Serious	Serious	Suspected	●●◯◯ Low
UPDRS-III	4 RCTs	SMD = −0.04 (−0.53~0.44)	Serious	Serious	Not Serious	Serious	Undetected	●◯◯◯ Very low

## Data Availability

The data analyzed in this study are publicly available in the cited articles.

## Supplementary Materials

The following supporting information can be downloaded at: https://www.mdpi.com/article/10.3390/brainsci16040359/s1.
